# Supplementary material for: Development of a constant pressure perfused ex vivo model of the equine larynx
Source: PLoS One. 2021 May 20;16(5):e0251530. doi: 10.1371/journal.pone.0251530 (PMC8136745; doi:10.1371/journal.pone.0251530)
Supplement: Attachment — Submitted filename: Response to Reviewers.docx [file pone.0251530.s002.docx]

Dear Dr. Maurizio Mandalá, dear Prof. Müller, dear reviewer 2,

thank you very much for sending us the incredibly helpful reviews!

Please find below the point-by-point answers to all comments raised by you.

1. **Journal Requirements**

Reg. 1) We apologize for the mistakes we made in the reference list. We corrected all citations.

Reg. 2) We adjusted the manuscript according to the style requirements by adding line numbers also to the first page, realigned the affiliations page accordingly and checked all captions (adjustments were necessary to fulfil the reviewers´ requirements).

Reg. 3) We adjusted the conflict of interest paragraph and, as required, copied it into the cover letter. It reads now: „The authors declare that no competing interests exists with respect to research, authorship and/or publication of this article.

This does not alter our adherence to PLOS ONE policies on sharing data and materials.“

Here is a detailed description towards the funding by Med-EL (company):

SO received a doctoral stipend for the duration of the project at Leipzig University. The funder provided financial support for research materials but did not have any additional role in the study design, decision to publish and manuscript preparation, data collection or analysis and no patents are pending.

2. **Comments raised by Reviewer 1 (Prof. Müller)**

First, we would like to explicitely thank you for your detailed and appreciative review of our manuscript.

1. Rev. 1 asked for a revision of the discussion towards the CAD contraction at the beginning/end of the experiment. We rewrote this by adding the abducted vocal fold and the perfusion duration (lines 294-295). This is indeed a central finding of this study, but as it already has a front row seat in the discussion, we don´t want to overinterpret this as it is the first study to report such data in a modest number of *ex vivo* larynges and we tend to judge our data as critical as possible.
2. The next point Rev. 1 recommends is to add information to the outlook about fresh blood perfusion in hindsight of oxygen supply and substrate nutrients. We were happy to do this and rearranged the text accordingly (lines 364-373).
3. Rev. 1 then asked to specify the duration of the electrical stimulation at the beginning and at the end of the experiment which was 8 s (minimum). We supplemented the manuscript with this (material and methods section, line 138).

We would like take the opportunity to comment on this factor of central importance to you: of course, one of the logical next steps would consist of stress testing the model by moving the vocal fold for a certain period of time/try different pulses etc. to determine the point where this model reaches its capacity. This will require quite a number of organs, an intelligent sub-grouping and will be complicated by the overall lacking data in this field for ex-vivo larynx perfusion towards the strength of the muscular answer, what is considered a weak answer (etcpp), where the cut-off is... Nevertheless, we absolutely agree that this is something highly desirable!

We intendedly used this test to prove our approach as resulting in a functional model and learned during our experiments what a hallmark this relatively simple test presents.

3. **Comments raised by Reviewer 2**
1. Rev.2 asked for a more detailed description of our set-up. We extended the caption of Fig 1 that explains the experimental set-up and inserted an additional schematic drawing of an equine larynx in order to better illustrate the catheterization.

The corresponding caption reads now (lines 120-134):

A: The perfusion system (technical drawing) consists of a closed circuit. Red color indicates the hot water circuit, blue represents the perfusion circuit, gassed with carbogen. For active perfusion, the arteries were catheterized, passive venous outflow drains into the perfusion tub. The gassing tubes (one in the supply tank 1 and one in the perfusion tub) are denoted with an asterisk. The perfusion flow was measured using a flowmeter (F). In the inset photograph, a dorsal view of the perfusion setup in situ can be observed. The larynx, immersed in perfusion solution, is placed on a larynx retainer to stabilize its position in the perfusion tub. Both cranial thyroid arteries were catheterized (intravenous catheter, 18 gauge). For pH-control, a pH-meter is added (marked with an arrow).

B: Schematic drawing of an equine larynx, lateral aspect. Relevant structures are the cricoarytenoideus dorsalis muscle [CAD, a)], the common carotid artery [b) running parallel to the external jugular vein], the catheterized arterial branch [c), cranial thyroid artery] followed by its ramification into the ascending pharyngeal artery (d) and the laryngeal branch (e). The latter two vessels nourish the CAD. f) Recurrent laryngeal nerve reaches the larynx as caudal laryngeal nerve.

Thank you very much for this helpful comment, we hope to have addressed it sufficiently.

2. The next comment by Rev. 2 addresses the electrical stimulation of the CAD. The contraction of the CAD can be seen by an abduction of the vocal fold. This was judged visually. We did not develop a comparable measurement approach for this and therefore it is stated descriptively in the text. To improve this passage, we refined the results accordingly (lines 225-226).
